# Energies and new technologies in pelvic and pelvic floor dysfunctions

**DOI:** 10.61622/rbgo/2025FPS6

**Published:** 2025-07-31

**Authors:** Adriana Bittencourt Campaner, Ana Lucia Ribeiro Valadares, Fabiene Bernardes Castro Vale, Letícia Maria de Oliveira, Lucas Schreiner, Lucia Alves Silva Lara, Lucia Costa-Paiva, Marair Gracio Ferreira Sartori, Neila Maria de Góis Speck, Zsuzsanna Ilona Katalin de Jármy Di Bella

**Affiliations:** Faculdade de Ciências Médicas Santa Casa de Misericórdia de São Paulo São Paulo SP Brazil Faculdade de Ciências Médicas, Santa Casa de Misericórdia de São Paulo, São Paulo, SP, Brazil.; Departamento de Tocoginecologia Universidade Estadual de Campinas Campinas SP Brazil Departamento de Tocoginecologia, Universidade Estadual de Campinas, Campinas, SP, Brazil.; Departamento de Ginecologia e Obstetrícia Faculdade de Medicina Universidade Federal de Minas Gerais Belo Horizonte MG Brazil Departamento de Ginecologia e Obstetrícia, Faculdade de Medicina da Universidade Federal de Minas Gerais, Belo Horizonte, MG, Brazil.; Departamento de Ginecologia Escola Paulista de Medicina Universidade Federal de São Paulo São Paulo SP Brazil Departamento de Ginecologia, Escola Paulista de Medicina, Universidade Federal de São Paulo, São Paulo, SP, Brazil.; Pontifícia Universidade Católica do Rio Grande do Sul Porto Alegre RS Brazil Pontifícia Universidade Católica do Rio Grande do Sul, Porto Alegre, RS, Brazil.; Departamento de Ginecologia e Obstetrícia Faculdade de Medicina de Ribeirão Preto Universidade de São Paulo São Paulo SP Brazil Departamento de Ginecologia e Obstetrícia, Faculdade de Medicina de Ribeirão Preto, Universidade de São Paulo, São Paulo, SP, Brazil.; Departamento de Tocoginecologia Universidade Estadual de Campinas Campinas SP Brazil Departamento de Tocoginecologia, Universidade Estadual de Campinas, Campinas, SP, Brazil.; Departamento de Ginecologia Escola Paulista de Medicina Universidade Federal de São Paulo São Paulo SP Brazil Departamento de Ginecologia, Escola Paulista de Medicina, Universidade Federal de São Paulo, São Paulo, SP, Brazil.; Departamento de Ginecologia Escola Paulista de Medicina Universidade Federal de São Paulo São Paulo SP Brazil Departamento de Ginecologia, Escola Paulista de Medicina, Universidade Federal de São Paulo, São Paulo, SP, Brazil.; Departamento de Ginecologia Escola Paulista de Medicina Universidade Federal de São Paulo São Paulo SP Brazil Departamento de Ginecologia, Escola Paulista de Medicina, Universidade Federal de São Paulo, São Paulo, SP, Brazil.

## Abstract

•Differences between laser, radiofrequency (RF) and microfocused ultrasound (MFU) energy-based devices (EBD).

•Indication of energy-based devices in genitourinary syndrome of menopause (GSM).

•Indication of energy-based devices in urinary incontinence (UI).

•What is known about the use of energy-based devices in sexual dysfunction (SD)?

•Indication of energy-based devices in vulvar lichen sclerosus (VLS).

**Recommendations**

- New technologies involving the use of high-power energies applied to the vagina and vulvar introitus should only be performed by trained gynecologists, which means theoretical knowledge about indications and complications and practical training using equipment registered with the National Health Surveillance Agency (Anvisa) in configurations previously established by the manufacturers.
- Before EBDs are applied to the vagina and vulva, patients should be advised that these are new technologies used as an alternative or complementary treatment to those considered first-line for dysfunctions such as GSM, stress urinary incontinence (SUI), VLS and some SDs associated with vulvovaginal atrophy.
- Patients should be advised that most studies favorable to the results of EBDs show subjective improvements and, to a lesser extent, objective improvements, and that some randomized studies have not confirmed differences between the use of EBDs and sham treatment in the evaluation of GSM and UI.
- Patients should be advised that the results obtained with EBDs are temporary, lasting approximately 12-18 months, but few studies have been conducted to assess the duration of the positive effects.
- For selected cases of well-diagnosed VLS in which the presence of neoplastic lesions has been ruled out and corticosteroid therapy is unresponsive, the use of EBDs following well-established protocols may be recommended.
- The concomitant use of EBDs with corticosteroid therapy may be an alternative for long-term VLS control by adjusting lower doses and the potency of corticosteroids.
- Patients should be advised regarding possible acute complications such as burns, vaginal discharge, urinary urgency and possible late effects associated with frequent repetition of procedures or excessive use of EBDs, such as vaginal fibrosis.
- Although the use of EBDs for functional and reparative purposes can be indicated, their use for exclusively aesthetic purposes in the intimate area should not be encouraged.

## Background

New technologies involving EBDs applied to the vagina and vulva have been used in the last decade for various gynecological conditions. Despite this fact, the literature data are still controversial regarding the long-term efficacy and safety of these therapies. One of the biggest current concerns is that the indiscriminate repetition of application of these energies leads to vaginal fibrosis and chronic pain. This document is based on systematic reviews regarding the use of the main EBDs in gynecological conditions that compromise health and quality of life (QoL). To date, the use of EBDs is not considered the gold standard of treatment for any of these indications by any scientific society.

### What are the most widely used EBDs in gynecology today?

The energies can be microablative (causing tiny lesions in the epithelium), such as CO_2_ laser, and microablative radiofrequency (RF) and microfocused ultrasound (MFU)(causes tiny lesions in deeper layers without reaching the epithelium), or non-ablative, such as non-ablative RF and Erbium laser. Energies using photobiomodulation are still little studied in gynecology.

### What are the mechanisms of action of these EBDs?

They act on the vaginal or vulvar epithelium through a thermal effect by activating heat shock proteins that improve tissue vascularization and stimulate the formation of collagen and elastin, leading to thickening of the epithelium layers with the formation of new papillae, increased glycogen production and reestablishment of the *Lactobacillus* population with production of lactic acid, restoring vaginal pH and trophism of the vaginal mucosa. So far, from a clinical point of view, no significant difference between the types of EBDs has been observed.

### What is known about the use of EBDs in genitourinary syndrome of menopause (GSM)?

Microablative and non-ablative therapies have been used to treat vaginal epithelial atrophy in GSM. Several scientific societies have raised concerns regarding the lack of robust evidence, since most studies with these technologies are prospective open-label with few randomized clinical trials.^(1)^ More recent studies and meta-analyzes have been reviewed and help to position the current evidence and safety of using these technologies.^(1-6)^

Some randomized clinical trials and systematic reviews using laser indicate that vaginal treatment can lead to significant improvements in clinical parameters, such as the Vaginal Health Index (VHI), which assesses elasticity, fluid volume, vaginal pH, epithelial integrity and moisture, and in the symptoms of vaginal dryness and dyspareunia.^(2-6)^

A recent meta-analysis observed that vaginal conjugated estrogens produced the best results, followed by CO_2_ laser and promestriene. The study concluded that CO_2_ laser is effective in the symptoms of GSM with few adverse events. However, confidence in these findings is low due to the risk of bias and the low number of clinical trials.^(7)^

The most recent systematic review, published in 2025, included studies of laser and RF for GSM. The main findings of the review indicate that CO_2_ laser compared with sham treatment may result in little or no difference in dysuria, dyspareunia, or QoL with a low level of evidence. Compared with vaginal estrogen, CO_2_ laser also showed little or no difference in dyspareunia, dryness, discomfort/irritation, dysuria, or QoL questionnaires, again with a low level of evidence. The effects of Erbium laser or RF on any outcome are uncertain, with a very low level of evidence. The studies reported few adverse events and no serious events. The authors suggest that although EBDs are promising, the current evidence is insufficient to confirm their efficacy compared to hormonal treatments or placebo.^(8)^

The use of non-ablative RF in GSM is even more recent and the number of clinical trials with this technology is scarce. In a recent study, the effect of non-ablative RF was similar to that of vaginal estrogen with improvement in clinical symptoms, VHI and vaginal pH, and superior to that of vaginal moisturizer, with improvement in some histomorphometric parameters, showing promising results.^(9)^

Recently, a clinical trial compared the effects of CO_2_ laser with microablative RF and vaginal promestriene. At the end of the study, the three treatments produced similar effects: reduction in vaginal pH and improvement in vulvovaginal symptoms and sexual function with no differences between the groups. Side effects were mild for both EBDs groups, mainly represented by vaginal discharge.^(10)^

In other national randomized studies comparing CO_2_ laser, microablative RF and vaginal estrogen using estriol in postmenopausal women and promestriene in breast cancer survivors, similar improvements in VHI and QoL questionnaires were found between these EBDs and vaginal estrogen therapy, even in cancer survivors using antiestrogens.^(11,12)^

Based on the clinical trials and systematic reviews available, energy therapies are promising, with the most robust evidence for CO_2_ laser compared to Erbium laser and RF. These technologies may be effective mainly in vaginal atrophy, although in a similar way to estrogen. They are particularly interesting for women who cannot use estrogen or for those who did not adhere to the use of vaginal estrogen or did not obtain a response with it. The incidence of complications is low when used by properly trained gynecologists.^(13)^

### What is known about the use of EBDs in Urinary Incontinence (UI)?

Urinary incontinence is a common condition in perimenopause and postmenopause that greatly interferes with women’s QoL. It is classified as SUI and urge UI (UUI), which frequently occurs in overactive bladder syndrome (OBS). There are different treatments, such as surgical treatments for SUI and medication for OBS, in addition to pelvic floor muscle therapy (PFMT). In the last decade, EBDs for GSM have empirically observed improvements in UI. Studies based on this observation have been published, not always with proper methodology, showing the possible role of EBDs in UI. In general, three applications of EBDs at monthly intervals are recommended, as is the case for GSM.

#### Stress urinary incontinence

To date, there are no published randomized studies between CO_2_ laser and Erbium laser, or between ablative and non-ablative RF for SUI. Energy-based devices such as MFU or the electromagnetic chair do not have long-term randomized follow-up studies or robust systematic reviews.^(13)^

A systematic review involving women with UI and/or pelvic organ prolapse showed small improvement, but included heterogeneous and low-quality studies. Adverse effects, such as mild pain and burning sensation were described, although without serious complications. The authors considered that the evidence on the effectiveness of laser in UI and genital prolapse were weak.^(14)^

In turn, a more recent systematic review with meta-analysis including randomized and cohort studies concluded that CO_2_ laser has a positive effect on UI, considering the results of the pad test and QoL questionnaires with up to one year of follow-up.^(15)^

On the other hand, based on heterogeneous studies and limited evidence, the meta-analysis involving six randomized studies evaluating comparative studies between EBDs and sham treatment did not find improvement in SUI. The application of CO_2_ laser did not lead to improvement in International Consultation on Incontinence Questionnaire – Short Form (ICIQ-SF) scores, the one-hour pad test, or the cure rate in follow-up up to six months. Given the insufficient number of studies, a meta-analysis for Erbium laser and RF could not be performed.^(16)^

In a systematic review of prospective studies, three with CO_2_ laser and one with Erbium laser, although good results in SUI were also observed in the short term, the role of therapy in the long term has not been established.^(17)^

Regarding Erbium laser, a randomized study with a sham-controlled group with laser for SUI stands out with only two applications at a six-week interval and six-month follow-up, and no differences were found between the groups in QoL questionnaires, 24-hour pad test and Likert satisfaction scale.^(18)^

Regarding non-ablative RF, a systematic review showed improvement in UI, chronic pelvic pain, pelvic floor muscle strength and sexual function. However, given the low quality of studies, more randomized studies are needed to formally recommend this therapy.^(19)^

Five randomized national studies conducted with different EBDs for women with SUI stand out, as presented below.

In a pioneering study, CO_2_ laser, microablative RF and sham were compared. After one year of follow-up, a subjective cure rate of 72.6% with CO_2_ laser was observed, as well as an objective cure of 45.2% (based on a negative stress test, a voiding diary without SUI episodes and a negative pad test). The results of microablative RF were 61.7% and 44.7%, respectively. The results of the sham group were 30% and 14%. All women were advised about behavioral therapies at the beginning of the study. In conclusion, the cure was similar between the EBD groups and both were superior to sham.^(20)^ Continent women were followed for up to four years; 50% of women treated with CO_2_ laser or microablative RF maintained urinary continence in the two-year follow-up after their respective treatments.^(21)^

In turn, in a randomized non-inferiority study, CO_2_ laser was not inferior to perineal physiotherapy performed twice a week in six months of follow-up using several QoL questionnaires.^(22)^

Comparing the Erbium laser with pelvic physiotherapy twice a week for three months in postmenopausal women with SUI, in the one-year follow-up using the pad test, 50% of cure was observed in physiotherapy and 56.25% after laser therapy with no difference between the therapies.^(23)^

When evaluating microablative RF with or without weekly PFMT for 12 weeks in women with SUI, similar improvements in the pad test and the female sexual function index (FSFI) were observed in both groups, as well as more significant improvements in QoL questionnaires with the combination of therapies.^(24)^

Regarding SUI, although there is still controversy regarding its indication, it is established that it has transient results, requiring periodic reapplications, in addition to uncertainty regarding late side effects.

#### Urgency incontinence – overactive bladder syndrome

While there are still doubts about the use of EBDs to treat SUI, their effect on UUI has been studied much less. A randomized national study observed that both microablative RF and sham improved QoL questionnaires related to urinary and vaginal symptoms, with no significant difference between them. There was also no improvement in sexual function or genital trophism. On the other hand, there was an improvement in endurance, resistance and rapid contractions of the pelvic floor in the group treated with microablative RF.^(25)^ Although to date, EBDs are not indicated in the treatment of UUI, many postmenopausal women experience an improvement in episodes of urinary urgency and frequency, which can improve other symptoms of OBS often present in GSM.

## What is known about the use of EBDs for the treatment of female sexual dysfunction (SD)?

Energy-based technology has been proposed as a therapy for SD, especially in women with GSM. However, the scientific robustness of these interventions is still a subject of debate.

In 2017, a systematic review involving women with GSM treated with CO₂ laser observed significant improvement in all domains of the FSFI, including desire, arousal, lubrication, orgasm, satisfaction and pain. In addition, there was a significant reduction in dyspareunia and an increase in sexual frequency. No relevant adverse effects were reported, and the subjective perception of sexual function also improved. Despite these promising findings, methodological limitations stand out, such as the absence of a control group, short follow-up time, and samples restricted to a few European centers.^(26)^

In 2022, a randomized clinical trial evaluated women with SD treated with vaginal CO₂ laser or Kegel exercises. The group treated with laser showed significant improvement in the lubrication domain from the sixth month onwards. However, FSFI scores remained below the normal cutoff point, indicating partial benefit on overall sexual function.^(27)^

In a prospective study using vaginal RF in three sessions, including women who survived gynecological or breast cancer and women in natural postmenopause, a significant improvement in FSFI scores was found in both populations, with an average increase from 14 to 27 points, demonstrating similar efficacy of RF regardless of the etiology of SD.^(28)^

Regarding sexual function in women with SUI, in a systematic review followed by meta-analysis including randomized studies after therapy with Erbium or CO_2_ laser, RF and electromagnetic chair, improvement in SUI and positive data were found in some domains of questionnaires on sexual function, even though there was no confirmation of improvement in sexual function in these patients.^(29)^

In 2024, a Brazilian study evaluated women with SUI, comparing the impact of CO₂ laser with PFMT. The laser showed superiority in the domains of orgasm, pain and total FSFI score, while physiotherapy promoted an improvement in sexual desire.^(23)^

Although encouraging, these findings should be interpreted with caution. The studies have demonstrated improvements in FSFI scores, but there is still a lack of sham-controlled clinical trials with sufficient methodological rigor to prove the efficacy and safety of energy-based therapies in the treatment of SD. Relevant variables, such as relational factors, age, body mass index and comorbidities must be controlled, otherwise the validity of the findings can be compromised. Furthermore, FSFI alone is not sufficient to make a diagnosis, and the inclusion of instruments that assess sexual satisfaction is essential. Future studies should consider these limitations.

## What is the role of energy use in Lichen Sclerosus Vulvaris (LSV)?

Linchen sclerosus vulvaris is a chronic inflammatory disease that affects the anogenital region, significantly impacting women’s QoL due to symptoms such as pruritus, pain and dyspareunia, in addition to anatomical sequelae. First-line treatment involves long-term application of high-potency topical corticosteroids, which, although effective, may have side effects and do not provide complete relief. Treatment with EBDs – the main ones being laser, fractional RF and MFU – is a promising alternative or adjuvant modality for LSV, demonstrating potential for improving symptoms, tissue trophism and QoL. Sequelae caused by LSV, such as introital stenosis, clitoral adhesions and hood fusion can be corrected using ablative energy, such as CO_2_ laser or RF, configured for surgical cutting.^(30-35)^

Studies suggest that the use of EBDs promotes greater reduction in pruritus, pain and dyspareunia in one to three months, compared with the use of topical corticosteroids.^(31)^ Some studies show improvement in clinical signs, such as improved skin elasticity and coloration and reduced sclerosis.^(34-36)^ Its use has been shown to improve QoL in refractory cases and it can be considered an alternative to corticosteroids during maintenance therapy.^(37,38)^

In terms of safety, EBDs are generally well tolerated. Adverse events are typically mild and transient, such as burning, hyperemia and edema with minor burns and blisters.^(34)^

The treatment can be applied to the vulva, perineum, and perianal region, sometimes including the vaginal canal. Protocols vary according to the type of EBD applied, but often involve three to five sessions at four to six-week intervals.^(33,34)^

Energy-based devices are contraindicated in cases of active infections in the treatment area, history of abnormal healing, and recent use of isotretinoin. Patients with autoimmune or immunosuppressed diseases should be evaluated with caution. Cases of lichen refractory to corticosteroid treatment that present clinically with thickened and exulcerated areas require prior biopsy to rule out the presence of a concomitant differentiated neoplasia.^(38,39)^

Energy-based devices show potential as valuable tools in the treatment of LSV, particularly for symptom relief and improvement of tissue quality. Although the evidence on its efficacy is still under study, there is growing data on its benefits, especially for CO_2_ laser, but more high-quality, long-term, randomized controlled clinical trials are needed to definitively establish its role and optimize treatment protocols. Future research should focus on direct comparisons with traditional treatments, identifying ideal treatment parameters, and on the potential benefits of combined therapies. Long-term safety and the impact on the risk of malignant transformation of lichen also justify further investigation.^(40)^

## Final considerations

The use of EBDs in GSM, SUI and LSV has a growing number of studies, although the main scientific societies do not yet consider their indications as well established. In OBS and SD, data are still in their infancy. There is undoubtedly a need for randomized, long-term, comparative studies between EBDs, in addition to establishing the precise indications for each of the conditions. There is no consensus regarding reapplications, and caution with the potential risks of stenosis or vaginal scarring is needed.
